# The evolving story of *Streptococcus gallolyticus*: classification, pathogenesis, role in human and animal disease, and laboratory diagnostics

**DOI:** 10.3389/fmicb.2026.1765252

**Published:** 2026-02-11

**Authors:** Veeraya Bamrung, Panchan Sitthicharoenchai

**Affiliations:** 1 Department of Population Health and Pathobiology, College of Veterinary Medicine, North Carolina State University, Raleigh, NC, United States; 2 Department of Veterinary Diagnostics and Population Animal Medicine, College of Veterinary Medicine, Iowa State University, Ames, IA, United States

**Keywords:** bacterial pathogenesis, colorectal cancer, gut microbiota, infective endocarditis, *Streptococcus bovis*/*Streptococcus equinus* complex, *Streptococcus gallolyticus*

## Abstract

*Streptococcus gallolyticus*, formerly known as *S. bovis*, belongs to the *Streptococcus bovis*/*Streptococcus equinus* complex (SBSEC). Besides being a part of the gut microbiome, this organism has gained interest due to its association with infective endocarditis and its strong correlation with colorectal cancer in humans. In veterinary medicine, systemic infection caused by *S. gallolyticus* has been reported in various animal populations, including porcine, ruminant, and avian species. Despite its clinical importance in humans and animals, two key challenges persist: the limited understanding of the pathogenesis due to its ubiquitous nature and inconsistencies in diagnostic laboratory reporting of the bacteria in SBSEC. This review summarizes the taxonomic characterization of the SBSEC, its clinical manifestations across species, current understanding of the bacterial pathogenesis, and the laboratory diagnostic assays used for its detection. We will further discuss the importance of SBSEC speciation and subspeciation, highlighting their distinct clinical implications and potential impact on human and animal health.

## Highlights

- *S. gallolyticus* (a member of SBSEC) is part of the gut microbiota and a significant pathogen in both humans (linked with IE and CRC) and animals (systemic infection in ruminants, pigs, and turkeys), with trending host predilections and differences of pathogenicity between each member of SBSEC.
- Frequent taxonomical revisions and inconsistent laboratory diagnostic reporting have complicated the epidemiology assessment of SBSEC and obscured the distinct clinical impact and prevalence of its specific species and subspecies.
- While *S. gallolyticus* is traditionally considered an opportunistic pathogen, its precise role in oncogenesis – whether as a “driver” or “passenger” or “both”– remains a subject of debate. Furthermore, this opportunistic label is challenged by findings, where certain strains serve as primary pathogens in turkeys.
- Future work on *S. gallolyticus* should focus on standardizing the laboratory diagnostic methods and implementing subspecies-specific reporting as a necessary foundation for robust epidemiological and genomic studies that can accurately assess the clinical risks of SBSEC for human and animal health.

## 1 Introduction

**Figure 1:**
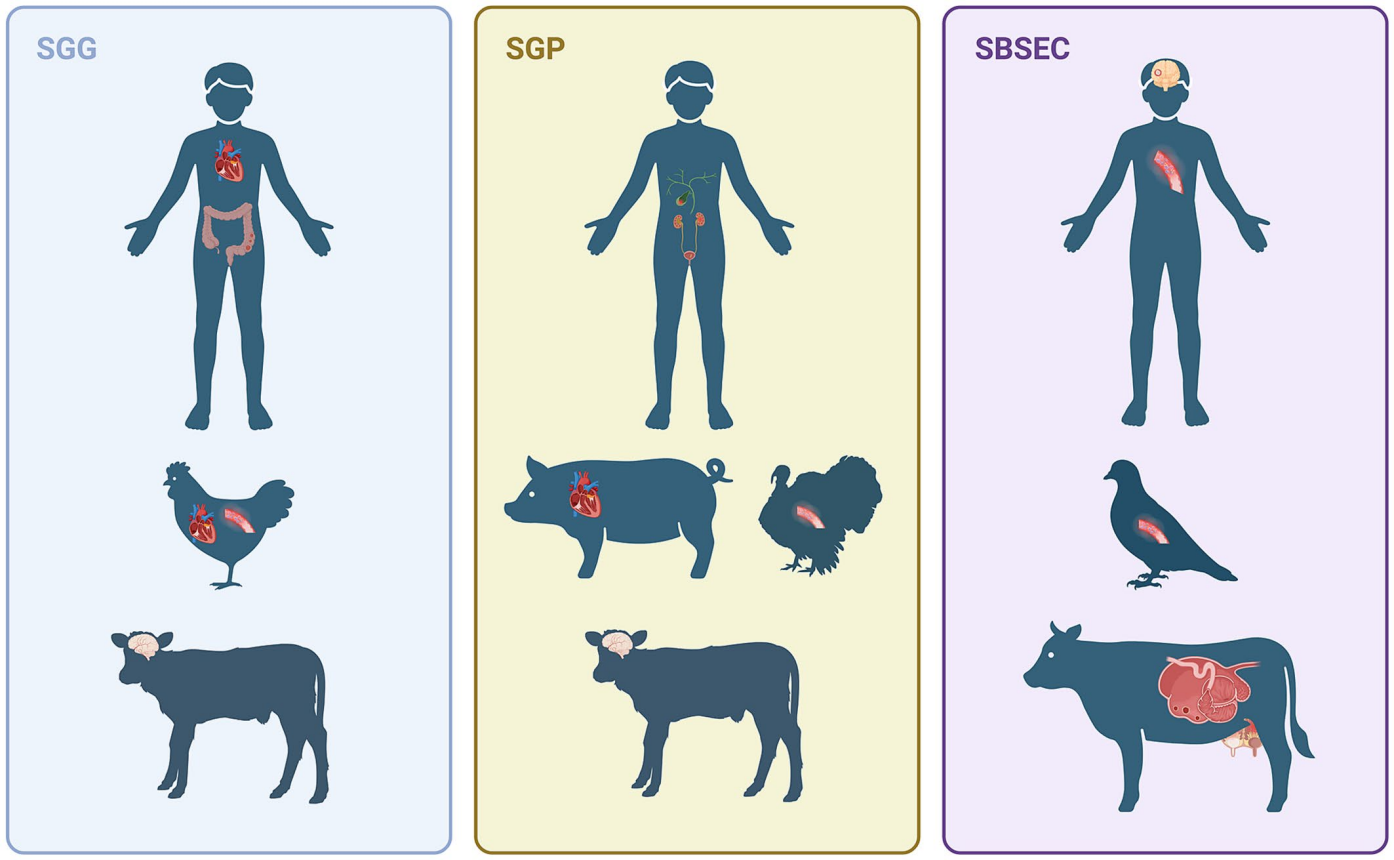
This figure summarizes the varied infections caused by *Streptococcus gallolyticus* in different hosts. *Streptococcus gallolyticus* subsp. *gallolyticus* (Sgg) is primarily linked to human endocarditis and colorectal cancer, as well as endocarditis and sepsis in chickens and meningitis in calves. *Streptococcus gallolyticus* subsp. *pasteurianus* (Sgp) is the predominant cause of human biliary and urinary tract infections, porcine endocarditis, turkey sepsis, and calf meningitis. Infections attributed to *Streptococcus bovis/Streptococcus equinus* complex (SBSEC) have also been reported, including human meningitis and sepsis, pigeon sepsis and rumen acidosis, and mastitis in ruminants; however, the specific species or subspecies was not indicated in these particular cases [Created in BioRender. Sitthicharoenchai, P. (2025) https://BioRender.com/r6m6g5b].

*Streptococcus gallolyticus* belongs to the *Streptococcus bovis*/*Streptococcus equinus* complex (SBSEC) and the *S. bovis* group (Schlegel et al., 2003). *S. gallolyticus* is part of the gut microbiota and an opportunistic pathogen, causing systemic infectious diseases and deaths in humans and animals (Figure 1). The strong link between *S. gallolyticus* and human infective endocarditis (IE), combined with the frequent bacteremia in colorectal cancer (CRC) patients, raises questions about its potential role in bacterial-induced cancer development. *S. gallolyticus* was not traditionally considered highly relevant in veterinary medicine. However, recent findings indicate a growing clinical significance. This includes emerging outbreaks of *Streptococcus gallolyticus* subsp. *pasteurianus* (Sgp) as a primary pathogen in turkey (Gray et al., 2023) and a causative agent of IE in pigs (Sitthicharoenchai et al., 2022). These prompt concerns about further understanding of bacterial pathogenesis and potential interspecies transmission, both animal-to-animal and animal-to-human. This review provides a comprehensive overview of *S. gallolyticus*, detailing its taxonomic classification, clinical presentation across various species, pathogenic mechanism, and current laboratory diagnostic methodologies. Furthermore, we will discuss the limitations of our understanding of *S. gallolyticus* and approach to fill the knowledge gaps.

Due to the high genomic diversity and the advent of modern molecular identification techniques, the taxonomy of *S. gallolyticus* has undergone several major reclassifications. To maintain clarity throughout this review, we use the broader term SBSEC when discussing findings from studies that refer to the bacteria as *S. bovis* or when specific species or subspecies differentiation within the complex is ambiguous.

## 2 Taxonomic classifications of *S. gallolyticus*

Advances in molecular and biochemical techniques have led to multiple reclassifications of SBSEC (Table 1). Historically, *S. gallolyticus* was categorized as *S. bovis* biotype I and II, which belong to the Lancefield Group D non-enterococcal streptococci (Orla-Jensen, 1919). This subclassification of *S. bovis* was based on the mannitol-fermenting group (biotype I) and the mannitol-negative fermenting group (biotype II) (Facklam, 1972; Parker and Ball, 1976). Biotype II was further subdivided into biotypes II/1 (non-fermenting) and II/2 (fermenting trehalose) based on DNA hybridization and trehalose fermentation results (Coykendall and Gustafson, 1985; Coykendall, 1989).

**Table 1:** Timeline of classifications and identification methods of SBSEC.

Year	1906-1919	1972-1976	1984	1985-1989	1990-2000	2002	2003
(Sub)species	*S. equinus*	*S. equinus*	Group 1	*S. equinus*	*S. equinus*	*S. equinus*	*S. equinus*
	*S. bovis* (Lancefield group D)	*S. bovis* biotype II	Group 1-4	*S. bovis* biotype II/1	*S. infantarius* subsp. *coli* (biotype II/1)	*S. lutetiensis*	*S. lutetiensis*
					*S. infantarius* subsp. *infantarius* (biotype II/1)	*S. infantarius*	*S. infantarius* subsp. *infantarius* (biotype II/1)
				*S. bovis* biotype II/2	*S. bovis* (biotype II/2)	*S. pasteurianus*	*S. gallolyticus* subsp. *pasteurianus* (biotype II/2)
		*S. bovis* biotype I		*S. bovis* biotype I	*S. gallolyticus* (biotype I)	*S. gallolyticus*	*S. gallolyticus* subsp. *gallolyticus* (biotype I)
					*S. macedonicus*	*S. macedonicus*	*S. gallolyticus* subsp. *macedonicus*
			Group 6 *S. alactolyticus*	*S. alactolyticus*	*S. alactolyticus*	*S. alactolyticus*	*S. alactolyticus*
			Group 5 *S. saccharolyticus*	*S. saccharolyticus*			
					*S. waius*		
Redesignation			Some *S. bovis* strains → *S. alactolyticus* Some *S. equinus* strains → *S. saccharolyticus*		*S. saccharolyticus* → *Enterococcus saccharolyticus* *S. caprinus = S. gallolyticus* *S. intestinalis = S. alactolyticus*	*S. waius* = *S. macedonicus*	
Identification Method(s)	Phenotypic characteristics Sugar fermentations Agglutination	Phenotypic characteristics Biochemical tests	DNA hybridization Biochemical tests	Phenotypic characteristics DNA hybridization Biochemical tests	Phenotypic characteristics DNA hybridization 16s, 23s ribosomal sequence Cell protein analysis Ribotyping Biochemical tests	Phenotypic characteristics DNA hybridization *sodA* sequence	Phenotypic characteristics DNA hybridization 16s ribosomal sequence Biochemical tests
Reference(s)	Andrewes and Horder (1906), Orla-Jensen (1919)	Facklam (1972), Parker and Ball (1976)	Farrow et al. (1984)	Coykendall and Gustafson (1985), Coykendall (1989)	Bentley et al. (1991), Bouvet et al., (1997), Brooker et al. (1994), Schlegel et al., (1999), Osawa et al. (1995), Schlegel et al. (2000), Sly et al. (1997), Tsakalidou et al. (1998), Vandamme et al. (1999)	Manachini et al. (2002), Poyart et al. (2002)	Schlegel et al. (2003)

This table summarizes the chronological evolution of the SBSEC taxonomy, including key *species*/*subspecies* designations, nomenclatural reassignments, and diagnostic methods used across decades. Taxonomic reclassifications, such as the renaming of *S. bovis* biotypes to *S. gallolyticus subspecies* and the reassignment of certain strains to other genera, were also documented. Symbols: → indicates reclassification or reassignment of the (sub) *species*, synonymous names or sub (*species*), leading to name change.

Farrow et al. (1984) further proposed a classification into 1–6 DNA groups based on the DNA–DNA hybridization, including two novel species: *S. saccharolyticus* (group 5) and *S. alactolyticus* (group 6). The term SBSEC was later proposed by the same research group based on their findings and a comparable serologic typing system and physiological reactions of *S. equinus* from other researchers (Kilpper-Bälz et al., 1982; Hillman et al., 1989; Andrewes and Horder, 1906; Smith and Shattock, 1962).

In the early 1990s, phylogenetic classification based on small subunit rRNA sequences became widely used for differentiating streptococcal species (Bentley et al., 1991). *S. saccharolyticus* was reclassified to the genus *Enterococcus* (Rodrigues and Collins, 1990). Combining the method with biochemical analyses led to the identification of novel (sub)species within the SBSEC, including *S. infantarius* (Bouvet et al., 1997), *S. macedonicus* (Tsakalidou et al., 1998), *S. waius* (Schlegel et al., 1999), *S. infantarius* subsp*. infantarius*, and *S. infantarius* subsp. *coli* (Schlegel et al., 2000). In 1995, Osawa et al. demonstrated tannase production in *S. bovis* biotype I. This discovery led to *S. bovis* biotype I being renamed as *S. gallolyticus* (Osawa et al., 1995). Another tannase-producing bacterium in goats, *S. caprinus*, was also reclassified under *S. gallolyticus* (Brooker et al., 1994; Sly et al., 1997).

Using DNA-DNA hybridization, *S. intestinalis* was considered a synonym of *S. alactolyticus* (Vandamme et al., 1999). A *sodA* gene-based phylogenetic interference was proposed as a classification system by Poyart et al. (2002), confirmed the synonymous assignments, and identified novel clusters: *S. lutetiensis* (previously *S. infantarius* subsp*. coli*) and *S. pasteurianus* (previously *S. bovis* biotype II/2) (Poyart et al., 2002). Based on DNA–DNA reassociation, *S. waius* is now considered as *S. macedonicus* (Manachini et al., 2002). The current classification of SBSEC was established by Schlegel et al. (2003) using a combination of DNA–DNA hybridization, 16S rDNA sequencing, and biochemical tests. This classification system includes three subspecies of *S. gallolyticus* (*S. gallolyticus* subsp. *gallolyticus* Sgg)*, S. gallolyticus* subsp. *macedonicus*, *S. gallolyticus* subsp*. pasteurianus* (Sgp), 1 subspecies of *S. infantarius* subsp. *infantarius* (Sii), and 3 separate species of *S. lutetiensis*, *S. alactolyticus*, and *S. equinus* (Schlegel et al., 2003).

## 3 *S. gallolyticus* link to colorectal cancer in humans and association with infective endocarditis

Members of the SBSEC are commensal bacteria belonging to the phylum *Firmicutes*, a major component of healthy human gut microbiota (Hou et al., 2022; Jandhyala et al., 2015). Several studies have revealed a shift in gut microbiome diversity in CRC patients, and the contribution of intestinal commensal bacteria to tumor development (Ahn et al., 2013; Boleij et al., 2011; Thomas et al., 2019; Kim and Lee, 2022; Fusco et al., 2024; Paduraru et al., 2025). Specifically, reports have demonstrated that fecal carriage of SBSEC is higher in CRC patients compared to healthy individuals (Klein et al., 1977; Périchon et al., 2022), suggesting a link between *S. gallolyticus* and CRC pathogenesis.

The clinical association between CRC and IE was first documented by McCoy and Mason (1951). The prevailing hypothesis suggests that tumor growth compromises the intestinal barrier, which allows opportunistic gut commensal bacteria to enter the bloodstream and subsequently colonize the heart valves (Keusch, 1974). This connection is supported by clinical data showing that 25 to 80% of patients with SBSEC bacteremia also have concomitant (Abdulamir et al., 2011). Furthermore, the reported association rate between SBSEC IE and CRC across various studies ranges from 18 to 62% (Abdulamir et al., 2011). While various species within the SBSEC bacteremia are associated with CRC, results from blood culture isolates show the strongest link with Sgg and Sgp, and to a lesser extent, Sii (Sánchez et al., 2014). Independent of its association with CRC, SBSEC has ranked among the top five causes of IE globally since the early 2000s (Vogkou et al., 2016; Öberg et al., 2022). Within this complex, Sgg has continued to be the predominant pathogen responsible for these infections (Öberg et al., 2022).

### 3.1 Proposed oncogenic mechanisms of *S. gallolyticus* and colorectal cancer

While single pathogens like *Helicobacter pylori* can directly induce tumorigenesis, *S. gallolyticus* fits the “driver-passenger” model, where multiple factors and several species of bacteria can contribute to tumor development (Tjalsma et al., 2012). There is an ongoing debate about whether *S. gallolyticus* plays an active role in the initiation of CRC through gene mutation (driver) or if its presence is simply a result of the tumor environment being suitable for its proliferation (passenger), or both (Avril and DePaolo, 2021).

As a “driver,” *S. gallolyticus* utilizes specific virulence factors to adhere and colonize the colonic mucosa. This persistent colonization stimulates chronic inflammation, eventually leading to DNA damage and tumor transformation. Several key virulence factors have been identified that facilitates in the adhesion and persistent colonization in the colonic epithelium and tumor cells. These include pilus loci (*pil1* and *pil3*) (Martins et al., 2016; Sillanpää et al., 2009), histone-like protein A (Boleij et al., 2009), and the Type VII secretion system (T7SS) (Taylor et al., 2021). Additionally, the Sgg pathogenicity-associated region (SPAR) appears essential for the function of T7SS, further promoting bacterial adhesion and colonization (Taylor et al., 2023). Following the adhesion and colonization, Sgg can induce the release of specific inflammatory cytokines (e.g., IL-1, COX-2, IL-8). These cytokines stimulate inflammation and cell proliferation via the Wnt/*β*-catenin pathway (Abdulamir et al., 2009; Kumar et al., 2017; Abdulamir et al., 2010; Moparthi and Koch, 2019). Consequently, chronic inflammation coupled with persistent Wnt activation can transform pro-oncogenic epithelial cells into cancer cells.

Furthermore, Sgg possesses unique characteristics that support the “passenger” role, allowing it to thrive and outcompete other gut commensal bacteria in the CRC microenvironment. It can produce bacteriocins such as gallocin and toxins from the LXG (leucine, any amino acid, glycine) family, that inhibit the growth of other commensal species (Aymeric et al., 2018; Taylor et al., 2021). Additionally, Sgg is bile-resistant, providing a competitive survival advantage in the bile-rich environment of the gut (Rusniok et al., 2010).

### 3.2 Role of pilus 1 in endocarditis development

The specific mechanism by which *S. gallolyticus* promotes IE is poorly understood and remains an understudied area. The basic development of IE requires a combination of endothelial injury and transient bacteremia, followed by bacterial adherence to the damaged site, and subsequent formation of vegetative growth. Whole-genome analysis of *S. gallolyticus* has highlighted the pilus and its role in adhering to heart tissues (Medrek and Barnes, 1962). *Pil1* has been shown to bind to collagen types I and IV, initiating bacterial attachment and promoting IE development (Danne et al., 2013). Collagen type I is abundant in the heart, providing clues to bacterial adhesion. Additionally, pil1 plays a role in biofilm formation, which has been shown to aid in the development of IE and help the bacterium evade the host’s immune response (Danne et al., 2013; Vollmer et al., 2010). The virulence genes *gtf*, *pilB*, and *fimB* were further identified in the study (Vollmer et al., 2010). Isenring et al. (2018) proposed a further mechanism by which Sgg enhances IE formation by disrupting the coagulation pathway related to *pil1*. Certain strains of Sgg were found to bind to FXII/PK via the pil1 protein. Such action leads to the aggregation and activation of FXII on the bacterial surface. This prolonged activated partial thromboplastin time and the release of bradykinin, potentially enhancing IE formation.

## 4 Other clinical forms of SBSEC infection in humans

The features of non-IE SBSEC infections have been reported sporadically; however, they have not been well defined. A 23-year retrospective study by Corredoira et al. (2014) investigated patients with biliary tract infections caused by SBSEC. It revealed that such infections resulting in cholangitis and cholecystitis are commonly associated with Sii and Sgp, accounting for 57 and 39% of 51 cases, respectively. The infection is often an ascending infection secondary to underlying blockage of the biliary tree (Lee et al., 2003; Corredoira et al., 2014). SBSEC is also associated with urinary tract infections (UTIs). The study, conducted from 1995 to 2012, shows that 45% of 88 patients with SBSEC bacteriuria were asymptomatic. The remaining patients display symptoms of lower UTIs (35%) or upper UTIs (20%) (Matesanz et al., 2014). Notably, Sgp is a dominant subspecies that infects urinary systems, and elderly women are predisposed to the infection (Clarridge et al., 2001; Fernández-Ruiz et al., 2010; Romero et al., 2011). Arthritis, osteomyelitis, and spondylodiscitis caused by SBSEC have been reported as complications associated with IE through septicemic spread (García-País et al., 2016). Moreover, arthritis can also be predisposed in patients with prosthetic joints, with Sgg responsible for 0.4% of 2,459 cases of prosthetic joint infection (Thompson et al., 2020). Lastly, neurological infections caused by SBSEC are sporadic, with meningitis reported in only 0.3–5% of cases and typically associated with other conditions. Other central nervous system (CNS) infections, such as abscesses and subdural empyema, are even less frequent (Cabellos et al., 1999; Sánchez et al., 2024; Baranda et al., 1985).

## 5 *S. gallolyticus* infection in animals

While SBSEC predominantly inhabits the gastrointestinal tract of ruminants and poultry, it can be detected in pigs, dogs, horses, and wildlife (Hodge and Sherman, 1937; Sillanpää et al., 2009; Maďar et al., 2021). Although sporadic opportunistic infections have long been recognized in various species, large-scale and cluster outbreaks of this bacterium cause significant economic losses in livestock and have raised concerns, highlighting the need for greater attention to it in veterinary medicine (Park et al., 2021; Sitthicharoenchai et al., 2022; Gray et al., 2023). The following section will detail the importance of SBSEC in various domestic animal species.

### 5.1 Rumen acidosis, mastitis, and systemic infection report in ruminants

*Streptococcus* spp. constitutes approximately 0.55% of the fecal microbiota in cattle (Dowd et al., 2008), with *S. bovis* as one of the major lactic acid-producing bacteria found in the digestive tracts of cattle, sheep, and other ruminants (Hardie, 1986). *S. bovis* can dominate the rumen microbiome when large amounts of soluble carbohydrates are provided (Hungate et al., 1952), resulting in excessive production of formic acid, acetate, and ethanol, and the development of ruminal acidosis (Russell and Baldwin, 1979). Furthermore, SBSEC has been one of the known causes of streptococcal mastitis in ruminants worldwide (Kabelitz et al., 2021; Kim et al., 2021; Iwanaga et al., 2022). The prevalence of SBSEC isolated in cases of mastitis is typically low and previously reported to be <1% of all streptococcal infections (Kabelitz et al., 2021). However, the emergence and higher prevalence of SBSEC-associated mastitis have been reported in certain regions, including Korea and Cambodia (Kim et al., 2021; Sophorn et al., 2025). SBSEC can also cause opportunistic systemic infection in ruminants. Specific subspecies, such as Sgp and Sgg, have been linked to suppurative meningitis-meningoencephalitis, causing neurological symptoms and mortality in calves with underlying predisposing factors, including failure of passive transfer and management issues (Seimiya et al., 1992; Sekizaki et al., 2008; Trotta et al., 2019).

### 5.2 Systemic infections and outbreaks in birds

SBSEC is found ubiquitously in the gastrointestinal tract of avian species (Garvie and Bramley, 1979). Opportunistic infection of *S. gallolyticus* resulting in septicemia has been reported in pigeons, waterfowls, turkey poults, and chickens (Devriese et al., 1990; De Herdt et al., 1994; Droual et al., 1997). However, during 2010 and 2013, widespread outbreaks of Sgp were reported in turkey flocks in Pennsylvania. The affected poults are at 2–3 weeks of age with clinical signs of sudden death (Saumya et al., 2014). Subsequent experimental challenge studies further confirmed that Sgp is a primary pathogen that causes septicemia in turkey poults (Gray et al., 2023). A shift in poult susceptibility to 1.5–2.5 weeks of age was noted in 2023 (Gray et al., 2024). Septicemia and meningitis were observed in goslings and ducklings, respectively (Barnett et al., 2008; Hogg and Pearson, 2009). In association with outbreaks of chicken disease, Sgg has been linked to endocarditis lesions and necrotic foci in the liver and spleen (De Herdt et al., 1994; Chadfield et al., 2007; Saumya et al., 2014). In pigeons, *S. gallolyticus* induces per-acute or acute streptococcal septicemia, reaching high mortality, especially in short-beak pigeons (De Herdt et al., 1994). Various predisposing factors have also been identified, including enteritis associated with viral, protozoal, and other bacteria. Additionally, nutritional deficiencies and specific conditions such as cage layer fatigue and dermatological lesions have been associated with streptococcal infections among avian species (Crispo et al., 2018).

### 5.3 Emerging cause of infective endocarditis in pigs

A retrospective study that analyzed 321 cases provides new insights into the common causes of endocarditis in USA domestic swine herds (Sitthicharoenchai et al., 2022). Sgp was recently described as the emerging causative agent contributing to 7.59% of swine valvular endocarditis. The reported cases of *S. gallolyticus*-associated endocarditis in swine were distributed across multiple states in the Midwest and Southeastern USA. The clinical signs include sepsis and sudden death in nursery to finisher pigs. The pathogenesis of Sgp infection of the heart valve remains unclear, though intestinal mucosal damage has been proposed as a potential predisposing factor.

## 6 Diagnostic laboratory identification of *Streptococcus gallolyticus*

SBSEC comprises a group of bacteria that can be readily grown on routine aerobic bacterial culture from clinical samples. The colony morphology on blood agar is small, gray, and exhibits *γ*-hemolysis. On microscopic examination, the bacteria are gram-positive, diplococci to chain-forming cocci. Common laboratory identification methods for SBSEC include biochemical testing, sequencing of the 16S rRNA and *sodA* genes (Poyart et al., 2002; Romero et al., 2011), as well as matrix-assisted laser desorption/ionization–time of flight mass spectrometry (MALDI-TOF MS) platforms. However, a single platform to accurately identify all SBSEC species and subspecies is not currently available. This difficulty arises from the high genetic conservation within the complex; for example, the 16S rRNA gene is nearly identical between some members of SBSEC (Jans et al., 2015). Recent data indicate that subspeciation of *S. gallolyticus* is most reliably achieved through *sodA* sequencing and the Vitek MS MALDI-TOF platform (Putnam et al., 2023). The ability to accurately identify SBSEC to the subspecies level is clinically important for the diagnosis of Sgg-associated IE and CRC, and its significance is increasing in veterinary medicine. Multiplex quantitative polymerase chain reaction (qPCR) has been developed to identify clinically significant SBSEC subspecies (Lopes et al., 2014). However, a limited number of samples were tested, and the sensitivity and specificity of this qPCR assay are unclear. Thus, there is a need for research efforts to improve and develop more robust diagnostic tools for the identification and classification of SBSEC. Until then, we recommend that diagnostic laboratories report isolates as SBSEC, followed by the species/subspecies or undetermined, accompanied by a statement outlining the limitations of the testing method used.

Multiplex antibody detection of Sgg pilus antigens has been developed in research settings for detecting preneoplastic stages of CRC. While results indicate that individuals with detectable Sgg antibody face a 40% increased risk of developing CRC within 10 years (Butt et al., 2018), the clinical utility is currently limited by the low assay sensitivity (16 to 43%) for early detection of CRC (Boleij et al., 2012). Therefore, these antibody results should be interpreted in conjunction with other robust diagnostic methods for CRC.

## 7 Discussion

*Streptococcus gallolyticus* infection is a multifaceted issue in both human and veterinary medicine. It is a recognized etiology of IE in humans and is strongly associated with CRC. Concurrently, it has become an emerging pathogen in various production animals, inflicting significant economic losses. Despite its established role in certain species, such as septicemia in turkeys, bovine mastitis, and swine IE, our understanding of the pathogenesis remains limited. Significant knowledge gaps remain regarding predisposing host conditions, the specific determinants of pathogenicity, and the extent of genomic variation among isolates from different animal species. While multiple virulence factors have been identified in human isolates of *S. gallolyticus*, their prevalence and function in animal clinical isolates have not been thoroughly investigated. Furthermore, the potential for zoonotic transmission of pathogenic strains and the challenge of antimicrobial resistance are of growing concern. Individuals with occupational exposure, such as farm workers and abattoir employees, or individuals who consume raw meat and dairy products, may be at a higher risk of infection.

Complicating this epidemiological assessment is the evolving taxonomy of the SBSEC, to which *S. gallolyticus* belongs. Studies have demonstrated that different species and subspecies within SBSEC displayed distinct host predilections and potential differences in pathogenicity. However, the frequent taxonomical revisions have affected the precise tracking of clinical prevalence and incidence data for individual species and subspecies of SBSEC. Furthermore, variations in diagnostic techniques employed by different laboratories for SBSEC identification and how the laboratory reports the results (as a complex or by species/subspecies) complicate the establishment of retrospective epidemiological data.

To fill this knowledge gap, future research should prioritize the standardization of the laboratory diagnostic methods, coupled with adopting the species- or subspecies-specific reporting as a foundational step that would significantly enhance our understanding of the clinical significance of individual SBSEC members. Such efforts should be supported by robust, ongoing epidemiological surveillance, with subsequent genomic sequence analysis and pathogenicity studies of both human and animal isolates, to further assess the interconnected risks to animal and public health.
